# In Vitro Glutamine Adaptability in Murine Liver- but Not Lung-Metastasizing Triple-Negative Breast Cancer Cells

**DOI:** 10.3390/cells15181630

**Published:** 2026-09-09

**Authors:** Marjorie Anne Layosa, Keigo Tomoo, Madeline P. Sheeley, Michael F. Coleman, Chaylen Andolino, Michael K. Wendt, Stephen D. Hursting, Dorothy Teegarden

**Affiliations:** 1 Department of Nutrition Science, College of Health and Human Sciences, Purdue University, West Lafayette, IN 47907, USA; mlayosa@purdue.edu (M.A.L.); ktomoo@purdue.edu (K.T.); madeline.sheeley@gmail.com (M.P.S.); candolin@purdue.edu (C.A.); 2 Purdue University Institute for Cancer Research, Purdue University, West Lafayette, IN 47907, USA; mwendt@purdue.edu; 3 Department of Nutrition, Gillings School of Public Health, University of North Carolina at Chapel Hill, Chapel Hill, NC 27599, USA; mcoleman@unc.edu (M.F.C.); hursting@email.unc.edu (S.D.H.); 4 Lineberger Comprehensive Cancer Center, University of North Carolina at Chapel Hill, Chapel Hill, NC 27599, USA; 5 Department of Basic Medical Sciences, College of Veterinary Medicine, Purdue University, West Lafayette, IN 47907, USA; 6 Nutrition Research Institute, University of North Carolina, Kannapolis, NC 28081, USA

**Keywords:** breast cancer, TNBC, metastasis, metabolic adaptation, glutamine, glutathione, metabolic plasticity, pyruvate carboxylase, organotropic metastasis

## Abstract

**Background:** Metabolic adaptability plays a critical role in supporting the growth and survival of metastatic breast cancer cells, and potentially supports site-specific metastasis. This study investigates the differential metabolism of glutamine and adaptability to varying glutamine levels of triple-negative breast cancer (TNBC) cells that metastasize to the lungs (metM-Wnt^Lung^) or liver (metM-Wnt^Liver^). **Methods:** The metastatic cell lines were exposed to varying in vitro glutamine concentrations (0.5, 2, and 4 mM). Cell viability, migration, ^14^C-glutamine uptake, mRNA abundance of metabolic enzymes, and ^13^C_5_-glutamine cellular flux, were measured. **Results:** At an intermediate level of in vitro glutamine supplementation (2 mM), metM-Wnt^Lung^ cells exhibited greater glutamine uptake, catabolic enzyme expression, and flux of glutamine-derived carbon into the TCA cycle compared with metM-Wnt^Liver^ cells. Despite this, metM-Wnt^Liver^ cells were more viable and migratory than metM-Wnt^Lung^ cells under both higher (4 mM) and lower (0.5 mM) glutamine levels, suggesting metabolic adaptability. Exposure to ammonia upregulated ammonia-assimilating enzymes in metM-Wnt^Liver^, but not metM-Wnt^Lung^ cells, plausibly mitigating ammonia toxicity in higher glutamine conditions. In glutamine-deprived conditions, the metM-Wnt^Lung^ cells maintained higher glutamine oxidation, but the metM-Wnt^Liver^ cells had higher total glutathione and GSH/GSSG ratios and enhanced resistance to oxidative stress, suggesting glutamate utilization toward glutathione synthesis. Additionally, metM-Wnt^Liver^ cells utilized glucose-derived carbons through pyruvate carboxylase (PC) to maintain TCA cycle activity, with elevated PC expression and M+3-labeled oxaloacetate enrichment to a greater extent than metM-Wnt^Lung^ cells. PC silencing in metM-Wnt^Liver^ cells enhanced cell viability under glutamine deprivation, which was reversed by inhibiting phosphoglycerate dehydrogenase (PHGDH, a key enzyme in de novo serine synthesis). We propose that PC activity compensates for low glutamine levels, including diverting glucose to adaptive pathways such as de novo serine synthesis. **Conclusions:** Overall, our findings demonstrate that metM-Wnt^Liver^ cells, but not metM-Wnt^Lung^ cells, exhibit glutamine-specific metabolic plasticity, characterized by the modulation of glutamine catabolism, enhanced ammonia metabolism and antioxidant defense, and support of glucose metabolism, which are associated with better cell survival and migration under glutamine excess and deprivation.

## 1. Introduction

Breast cancer is the most common cancer among women, accounting for 31% of all female cancers, and metastases are responsible for the majority of deaths from the disease [1,2]. Breast cancer is a heterogeneous disease with distinct subtypes, each exhibiting a risk of metastasizing to specific distant organs, such as the bones, lungs, liver, and brain [3,4]. Among these subtypes, triple-negative breast cancer (TNBC) is particularly aggressive and associated with a poorer prognosis [5]. Notably, TNBC has the highest frequency of metastasis to the lungs (~45%) among all subtypes, and a higher frequency of liver metastasis (~40%) compared to a hormone receptor-positive subtype [3]. A key determinant of successful metastasis is the ability of tumor cells to adapt metabolically to the unique nutrient environments of metastatic sites [4,6,7]. The distinct metabolic features of the lungs and liver present significant challenges for metastasizing cancer cells [4,6,8], and metabolic reprogramming may enable tumor cells to overcome these barriers, sustain their energy demands, and thus enhance their metastatic potential to specific sites [4,9].

The role of glucose in supporting tumorigenesis and tumor progression is well known, and therapies targeting glucose metabolism are well studied [10]. However, the role of amino acid metabolism in cancer has received substantial attention in recent years [11]. Glutamine, the most abundant amino acid in the body [12,13], is an additional important growth-supporting substrate that contributes not only carbon but also reduced nitrogen for the de novo biosynthesis of a number of diverse nitrogen-containing compounds such as non-essential amino acids and nucleotides [14]. Although glutamine is a non-essential amino acid, transformed cells have been shown to consume glutamine at a rate that exceeds endogenous synthesis, making glutamine essential, at least in some circumstances of rapid growth [8,15]. Glutamine metabolism, through conversion to α-ketoglutarate in the tricarboxylic acid (TCA) cycle, allows replenishment of TCA metabolites when energy production from aerobic glycolysis is insufficient [15,16]. Glutamine also provides essential precursors for the biosynthesis of reduced glutathione, a major antioxidant against oxidative stress [13,15]. Further, glutamine plays a role in immune modulation by regulating T cell proliferation and activation, which are important for anticancer immune response [15]. Together, these diverse functions underscore glutamine’s critical role in supporting tumor growth and survival, highlighting its potential as a therapeutic target in cancer metabolism.

Several studies suggest that TNBC cells are particularly “addicted” to glutamine as compared to other subtypes [11,17,18]. TNBC cells are often characterized by the overexpression of glutaminase (GLS), the initial and rate-limiting step in glutaminolysis that converts glutamine to glutamate, resulting in enhanced glutamine catabolism [11,17] and sensitivity to drugs targeting glutamine-related pathways [18]. However, there are reports that some patients with TNBC exhibit de novo resistance to glutamine pathway-targeting drugs, potentially involving metabolic rewiring to utilize other energy sources such as glucose and fatty acids when glutamine is limited [18,19]. This underscores the metabolic complexity of cancer and suggests that the ability of TNBC cells to adapt to varying glutamine levels may serve as a potential therapeutic target, particularly since there is a lack of effective molecular targets for TNBCs [11].

Glutamine metabolism varies between organs and health conditions. For example, both the lungs and liver can produce glutamine, but the liver shifts to consuming glutamine under catabolic conditions [8,15]. Although the liver can both synthesize and degrade glutamine, it contributes minimally to plasma glutamine levels, with its activity regulated by hepatocyte heterogeneity, blood pH and ammonia levels [8]. Given the distinct glutamine metabolism in lungs and liver [8] and the risk of TNBC cells to metastasize to these organs [3], the ability and underlying mechanisms of metastatic TNBC cells to adapt to varying glutamine levels is a critical question and remains unclear.

In this study, we investigated glutamine metabolism and adaptive responses to varying in vitro glutamine levels in TNBC cells that preferentially metastasize to the lungs (metM-Wnt^Lung^ cells) or liver (metM-Wnt^Liver^ cells). Understanding how aggressive TNBC cells adapt to environmental stresses, such as glutamine availability, may uncover potential therapeutic strategies to hinder metastatic progression to these distinct sites. We hypothesize that metM-Wnt^Lung^ cells and metM-Wnt^Liver^ cells exhibit distinct glutamine metabolic profiles, which may support their ability to metastasize to the lungs and liver, respectively.

## 2. Methodology

### 2.1. Chemicals and Antibodies

Glutaminase inhibitor 968 (GLS-968), NCT-503 (phosphoglycerate dehydrogenase inhibitor), and dimethyl sulfoxide (DMSO) were purchased from Sigma-Aldrich (St. Louis, MO, USA). Glutathione ethyl ester was purchased from Cayman Chemical (Ann Arbor, MI, USA).

### 2.2. TNBC Cell Lines

Murine metM-Wnt^Lung^ and metM-Wnt^Liver^ cell lines were generated as reported [20,21]. In brief, these unique metastatic metM-Wnt^Lung^ and metM-Wnt^Liver^ cell lines were generated through serial passaging of our previously described, nonmetastatic M-Wnt TNBC cell line [20,21]. We used cells between passages of 5–20, and these cells are used in our in vivo study where they demonstrate preferential metastasis to either lung or liver. Mycoplasma testing was not performed on the cell lines used in this study.

### 2.3. Constitutive PC Silencing in metM-Wnt^Liver^ Cells

PC was suppressed in metM-Wnt^Liver^ cells using lentivirus, generated by the transfection of HEK293T cells with psPAX2 (Addgene, Watertown, MA, USA), pMD2.G (Addgene, Watertown, MA, USA), and Pcx-targeting shRNA purchased from Genecopoeia (CCCACAACTTCAACAAGCTCT), or a scramble control (GCTTCGCGCCGTAGTCTTA) and selected using hygromycin (500 µg/mL).

### 2.4. Cell Culture

The cell lines were maintained in modified Dulbecco’s Modified Eagle Medium (DMEM) containing 5 mM glucose, 2 mM glutamine, 10 mM HEPES, 3.7 g/L sodium bicarbonate, without sodium pyruvate, supplemented with 10% (*v*/*v*) fetal bovine serum (FBS) and 1% (*v*/*v*) penicillin–streptomycin antibiotic mix (Gibco, Billings, MT, USA), and incubated at 37 °C with a humidified 5% CO_2_ atmosphere. For experiments involving varying glutamine availability, cells were exposed either to a higher glutamine level (4 mM), a concentration commonly reached in serum or intracellular pools, or to a lower glutamine level (0.5 mM), selected based on physiological extracellular glutamine concentrations of ~0.6–0.9 mM.

### 2.5. Gene Expression

Cells (3.6 × 10^3^ cells/cm^2^) were seeded and allowed to attach overnight. The following day, media was changed into specified glutamine-defined levels for 48 h. Following incubation, RNA was extracted using TRI Reagent (Molecular Research Center, Cincinnati, OH, USA) following the manufacturer’s instructions. mRNA was then reverse transcribed using MMLV reverse transcriptase (Promega, Madison, WI, USA) and subsequently amplified with LightCycler 480 SYBR Green I Master Mix (Roche, Indianapolis, IN, USA). Relative mRNA expression levels were determined using the comparative C_T_ method, as previously described [22]. Primer sequences for qRT-PCR are provided in Table 1.

### 2.6. ^14^C Glutamine Cellular Uptake

Cells (3.6 × 10^3^ cells/cm^2^) were seeded and allowed to attach overnight. The following day, media was changed into specified glutamine-defined levels for 48 h. After the incubation period, medium was changed and L-[^14^C(U)]-glutamine (0.25 µCi/mL) (American Radiolabeled Chemicals, Inc., St. Louis, MO, USA) added and incubated at 37 °C for 10 min. Cells were subsequently washed twice and collected using cold phosphate-buffered saline (PBS, 137 mM NaCl, 2.7 mM KCl, 10 mM phosphate buffer). The ^14^C-labeled cell lysate was quantified using a Tri-Carb 5110TR 110 V Liquid Scintillation Counter (PerkinElmer, Waltham, MA, USA). At the same time, separate cell culture dishes were incubated with 0.1N NaOH overnight, and protein was quantified using a Pierce™ bicinchoninic acid (BCA) protein assay kit per the manufacturer’s instructions (ThermoFisher, Waltham, MA, USA). Radioactivity was expressed as DPMs normalized to protein.

### 2.7. ^13^C Metabolic Flux Analysis

Cells (3.6 × 10^3^ cells/cm^2^) were seeded and allowed to attach overnight. The following day, media was changed into specified glutamine-defined levels for 48 h. After incubation, media was replaced with fresh media containing the indicated [U-^13^C]glutamine or glucose, in separate experiments, for 2 h at 37 °C. Sample harvest and derivatization were conducted as reported [23]. Briefly, dishes were rapidly washed with cold 1× PBS, scraped, and collected using preheated (70 °C) 70% ethanol in water (*v*/*v*). Cell extracts were vortexed, heated at 95 °C for 5 min, and chilled on ice for 5 min. Supernatants were collected after centrifugation at 14,000× *g* for 5 min, and evaporated to dryness with nitrogen gas. Dried samples were derivatized with 2% methoxylamine hydrochloride in pyridine solution (*wt*/*vol*) and N-tert-butyldimethylsilyl-N-methyltrifluoroacetamide with 1% (*wt*/*wt*) tert-butyldimethylchlorosilane. Samples were analyzed with gas chromatography mass spectrometry (Thermo TSQ 8000 triple quadrupole mass spectrometer coupled with a Thermo Trace 1310 gas chromatography, ThermoFisher, Waltham, MA, USA). Metabolic ^13^C tracing was determined by dividing the total peak area of the labeled metabolite of interest to total peak area of all its isotopologues. Data is expressed as % of total pool following IsoCor correction [24].

### 2.8. Cell Viability

Cells (4.4 × 10^3^ cells/cm^2^) were seeded and allowed to attach overnight. The following day, media was changed into specified glutamine-defined levels with or without specified treatments for 48 h. Following incubation, the media was removed and replaced with 1× (0.5 mg/mL) 3-(4,5-dimethylthiazol-2-yl)-2,5-diphenyltetrazolium bromide (MTT) (Sigma-Aldrich, St. Louis, MO, USA) in serum-free media. Plates were incubated for 2 h at 37 °C, crystals dissolved in DMSO, and absorbance determined at 570 nm with a Biotech spectrophotometer. Viability is expressed as the relative absorbance readings. As an orthogonal method to validate viability results from the MTT assay, we also performed trypan blue exclusion tests of cell viability under varying glutamine levels [25].

### 2.9. Transwell Migration Assay

Cells (1.3 × 10^4^ cells/cm^2^) resuspended in a FBS-free culture medium with indicated treatments were seeded in trans well inserts (8 μm pore size, 24-well plate) (Corning, NY, USA). The lower chamber contained the culture medium with 10% FBS as a chemoattractant. After 17 h, migrated cells on the lower side of the membrane were fixed with methanol and stained with crystal violet for counting. Non-migrated cells on the upper surface of the membrane from one representative insert per treatment were also counted to account for cell proliferation. Random fields per well were imaged, and migration was expressed as % migration relative to total cells (migrated plus non-migrated cells).

### 2.10. Ammonia Quantification

Cells (3.6 × 10^3^ cells/cm^2^) were seeded and allowed to attach overnight. The following day, the media was changed into specified glutamine-defined levels for 48 h. Ammonia levels in the cell culture media were measured using an ammonia assay kit (Abcam, #ab83360, Waltham, MA, USA). In brief, metastatic cells were exposed to 4 mM glutamine and cell culture media were harvested at 48 h. Ammonia concentrations were determined using a standard curve prepared as per the manufacturer’s instructions. Absorbance was measured at 570 nm, and results were expressed as nmoles/mL, normalized to trypan blue cell count.

### 2.11. Glutathione Quantification

Cells (3.6 × 10^3^ cells/cm^2^) were seeded and allowed to attach overnight. The following day, media was changed into specified glutamine-defined levels for 48 h. Total and oxidized glutathione (GSH + GSSG and GSSG, respectively) levels were determined using colorimetric assay (MedChemExpress, HY-K0311, Monmouth Junction, NJ, USA) following the manufacturer’s instructions. In brief, total glutathione was measured in extracted cell supernatant by enzymatically reducing oxidized glutathione (GSSG) to reduced glutathione (GSH) using the enzyme glutathione reductase. The resulting GSH reacts with Ellman’s Reagent (DTNB) and absorbance was assessed at 412 nm. GSSG was measured by masking GSH in the sample using 1-methyl-2-vinylpyridinium triflate. The sample was treated with glutathione reductase and DTNB to convert GSSG to GSH, which was subsequently detected by measuring absorbance at 412 nm. Absorbance was recorded 15 times at one-minute intervals. Glutathione concentrations were calculated based on the slope (K) of the standard curve generated by linear regression of absorbance values from known standards. GSH levels were calculated using the formula: GSH = (GSH + GSSH) − GSSG × 2, and the GSH/GSSG ratio was determined. Cell pellets were incubated with 0.1N NaOH overnight, and the protein was quantified using the BCA protein assay kit. Results were normalized to protein and expressed as μM/mg protein.

### 2.12. Statistical Analysis

Quantitative data are presented as mean ± standard error of the mean (SEM) from three or more biological replicates. Statistical analyses were performed using a two-tailed Student’s *t*-test or two-way ANOVA, as indicated. Bonferroni’s multiple comparisons test was used for post hoc analysis following two-way ANOVA. Data visualization was conducted with GraphPad Prism 10.5.0. A *p*-value of <0.05 was considered statistically significant.

## 3. Results

### 3.1. Lung- and Liver-Metastasizing Breast Cancer Cells Differentially Metabolize Glutamine

**Figure 1:**
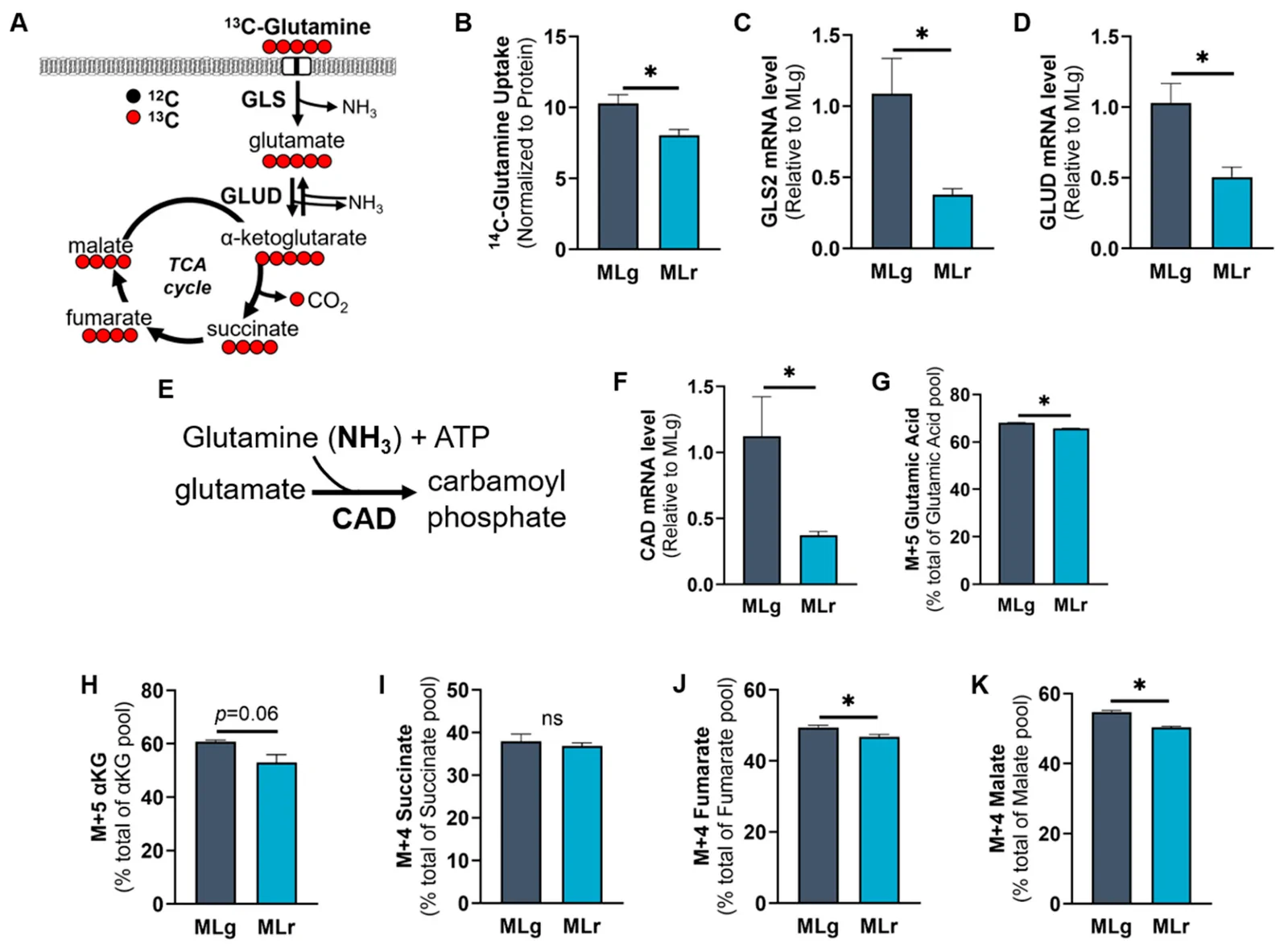
Glutamine metabolism into the tricarboxylic acid cycle. (**A**) Schematic diagram of ^13^C_5_-glutamine flux into tricarboxylic acid cycle intermediates. (**B**) ^14^C-glutamine uptake normalized to protein. (**C**) mRNA abundance of glutaminase 2 (GLS2), (**D**) glutamate dehydrogenase (GLUD), and (**F**) carbamoyl-phosphate 2 (CAD). mRNA levels were normalized to 18S and expressed relative to the met-MWnt^Lung^ levels. (**E**) Schematic diagram of utilization of glutamate by enzyme carbamoyl-phosphate 2 (CAD). (**G**) ^13^C_5_-glutamine incorporation into M+5-labeled glutamic acid, (**H**) M+5-labeled α-ketoglutarate (α-KG), (**I**) M+4-labeled succinate, (**J**) M+4-labeled fumarate, and (**K**) M+4-labeled malate. N = 4 biological replicates for all analyses. MLg = metM-Wnt^Lung^; MLr = metM-Wnt^Liver^. Asterisk indicates statistical significance (* *p* < 0.05, Student’s *t*-test); ns—not significant.

The metabolic fate of glutamine in TNBC cells that metastasize to the lungs compared to liver remains unclear; therefore, we assessed glutamine metabolism (Figure 1A) under an intermediate concentration of in vitro glutamine supplementation (2 mM). Results showed that ^14^C-glutamine uptake was significantly higher in the metM-Wnt^Lung^ cells, by 26%, compared to metM-Wnt^Liver^ cells (Figure 1B). Consistent with this, the mRNA abundances of glutamine-catabolizing enzymes, glutaminase 2 (*Gls2*, liver isoform) and glutamate dehydrogenase (*Glud*), were higher in metM-Wnt^Lung^ cells compared to metM-Wnt^Liver^ cells (Figure 1C,D). Likewise, mRNA levels of glutamate-utilizing enzyme carbamoyl-phosphate synthetase 2 (*Cad*) were higher in metM-Wnt^Lung^ cells (Figure 1E,F). In addition, intracellular ^13^C_5_-glutamine flux revealed that the metabolites such as M+5-labeled glutamate, M+4-labeled fumarate and M+4-labeled malate were significantly enriched in metM-Wnt^Lung^ cells compared with metM-Wnt^Liver^ cells (Figure 1G,J,K), with a similar trend (*p* = 0.06) with M+5-labeled α-ketoglutarate (Figure 1H). These results indicate that metM-Wnt^Lung^ cells have higher glutamine uptake, utilization, and incorporation into the TCA cycle compared to the metM-Wnt^Liver^ cells.

### 3.2. Liver-Metastasizing TNBC Cells Are More Adaptable to High Glutamine Levels than Lung-Metastasizing TNBC Cells

**Figure 2:**
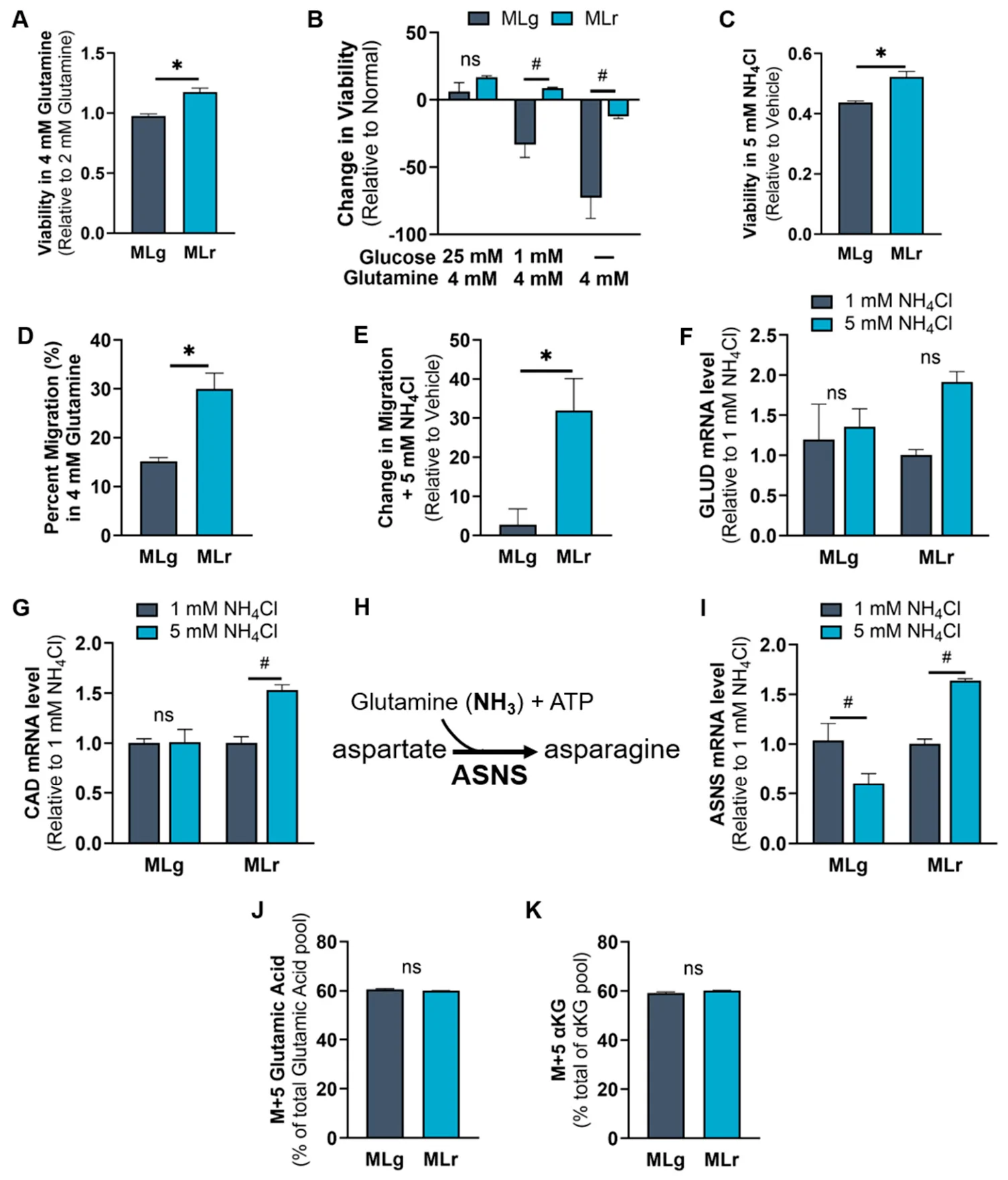
Adaptability to higher glutamine concentrations. (**A**) Viability of the cell lines after treatment with 2 mM or 4 mM glutamine for 48 h. (**B**) Change in viability of the cell lines after treatment with low (1 mM or 0 mM) or high (25 mM) glucose with 4 mM glutamine; no interaction effect. (**C**) Viability of the cell lines after treatment with 5 mM NH_4_Cl for 48 h relative to vehicle (water). (**D**) Percent migration (relative to total cells) in cells treated with 4 mM glutamine. (**E**) Change in migration (relative to vehicle with 2 mM glutamine) in cells treated with 2 mM glutamine and 5 mM NH_3_Cl for 17 h. (**F**) mRNA abundance of glutamate dehydrogenase (GLUD; no interaction effect), (**G**) carbamoyl-phosphate synthetase (CAD; interaction effect: *p* = 0.0096), and (**I**) asparagine synthetase (ASNS; interaction effect: *p* = 0.0002) after treatment with 1 or 5 mM NH_4_Cl for 48 h. mRNA levels were normalized to 18S and expressed relative to the 1 mM NH_4_Cl levels. (**H**) Schematic diagram of utilization of ammonia by enzyme asparagine synthetase (ASNS). ^13^C_5_-glutamine incorporation into (**J**) M+5-labeled glutamic acid and (**K**) M+5-labeled α-ketoglutarate (α-KG) at 4 mM glutamine. N = 5 biological replicates for viability assay; N = 4 biological replicates for migration, gene expression, and metabolic flux analyses. MLg = metM-Wnt^Lung^; MLr = metM-Wnt^Liver^ Asterisk indicates statistical significance (* *p* < 0.05, Student’s *t*-test); pound sign indicates statistical significance (# *p* < 0.05, Bonferroni’s multiple comparisons test); ns—not significant.

To investigate whether cellular adaptation to high glutamine levels differs between lung- and liver-metastasizing TNBC cells, cells were exposed to a higher glutamine concentration (4 mM) for 48 h. The metM-Wnt^Liver^ cells significantly increased viability by 18% at high glutamine concentrations, whereas high glutamine had no significant effect on the metM-Wnt^Lung^ cells, as compared to viability in 2 mM glutamine (Figure 2A, MTT assay). Consistent with these results, trypan blue cell counting showed significantly higher proliferation in metM-Wnt^Liver^ cells (99.1%) than metM-Wnt^Lung^ cells (90.8%) under high glutamine levels. Similarly, the metM-Wnt^Liver^ cells were more viable than the metM-Wnt^Lung^ cells when exposed to high glutamine with lower levels of glucose (1 mM and 0 mM; no interaction effects) (Figure 2B). These results suggest that the metM-Wnt^Liver^ cells are more adaptable to sustain viability under higher glutamine levels than the metM-Wnt^Lung^ cells.

### 3.3. Liver-Metastasizing TNBC Cells, but Not Lung-Metastasizing TNBC Cells, Utilize Ammonia and Are More Resistant to Ammonia at Higher Glutamine Levels

To investigate the metabolic mechanisms underlying adaptation to high glutamine, metastatic cells were exposed to ammonia, a by-product of glutamine catabolism. Results demonstrate that the metM-Wnt^Liver^ cells had a higher viability, by 8.5%, as compared to metM-Wnt^Lung^ cells after ammonia exposure (NH_4_Cl, 5 mM) (Figure 2C). The metM-Wnt^Liver^ cells also demonstrated higher migration under both high glutamine levels and ammonia exposure (Figure 2D,E). Although there is an increasing trend, mRNA abundance of enzymes capable of assimilating ammonia such as *Glud* was not significantly higher in metM-Wnt^Liver^ cells as compared to metM-Wnt^Lung^ cells upon high ammonia exposure (Figure 2F). Assimilation of ammonia via GLUD can result in the production of glutamate (Figure 1A). Interestingly, enzymes that utilized glutamine from glutamate such as *Cad* and asparagine synthetase (*Asns*) were upregulated in metM-Wnt^Liver^, but not in metM-Wnt^Lung^ cells upon ammonia exposure (Figure 2G–I). Further, the significant interaction effects indicate that the metastatic cell lines respond to ammonia levels differently, where an increase in *Cad* and *Asns* upon higher ammonia treatment was significantly higher in metM-Wnt^Liver^ cells. Under higher glutamine conditions, ^13^C_5_-glutamine tracing revealed the comparable enrichment of M+5-labeled glutamate and M+5-labeled α-ketoglutarate across metastatic cell lines (Figure 2J,K), suggesting similar ammonia production from glutamine catabolism through GLS and GLUD. Interestingly, extracellular ammonia levels, after exposure to higher glutamine levels, were significantly lower in the metM-Wnt^Liver^ cells (222.0 ± 4.6 nmoles/mL) than metM-Wnt^Lung^ cells (250.2 ± 2.9 nmoles/mL), suggesting greater metabolism of ammonia in the metM-Wnt^Liver^ cells and supporting the intracellular results. These findings indicate that metM-Wnt^Liver^ cells exhibit greater adaptability to elevated glutamine levels, associated with the upregulation of ammonia-assimilating enzymes that may mitigate ammonia toxicity and sustain glutamine catabolism under higher glutamine conditions.

### 3.4. Liver-Metastasizing TNBC Cells Are More Adaptable to Low Glutamine Levels than Lung-Metastasizing TNBC Cells

**Figure 3:**
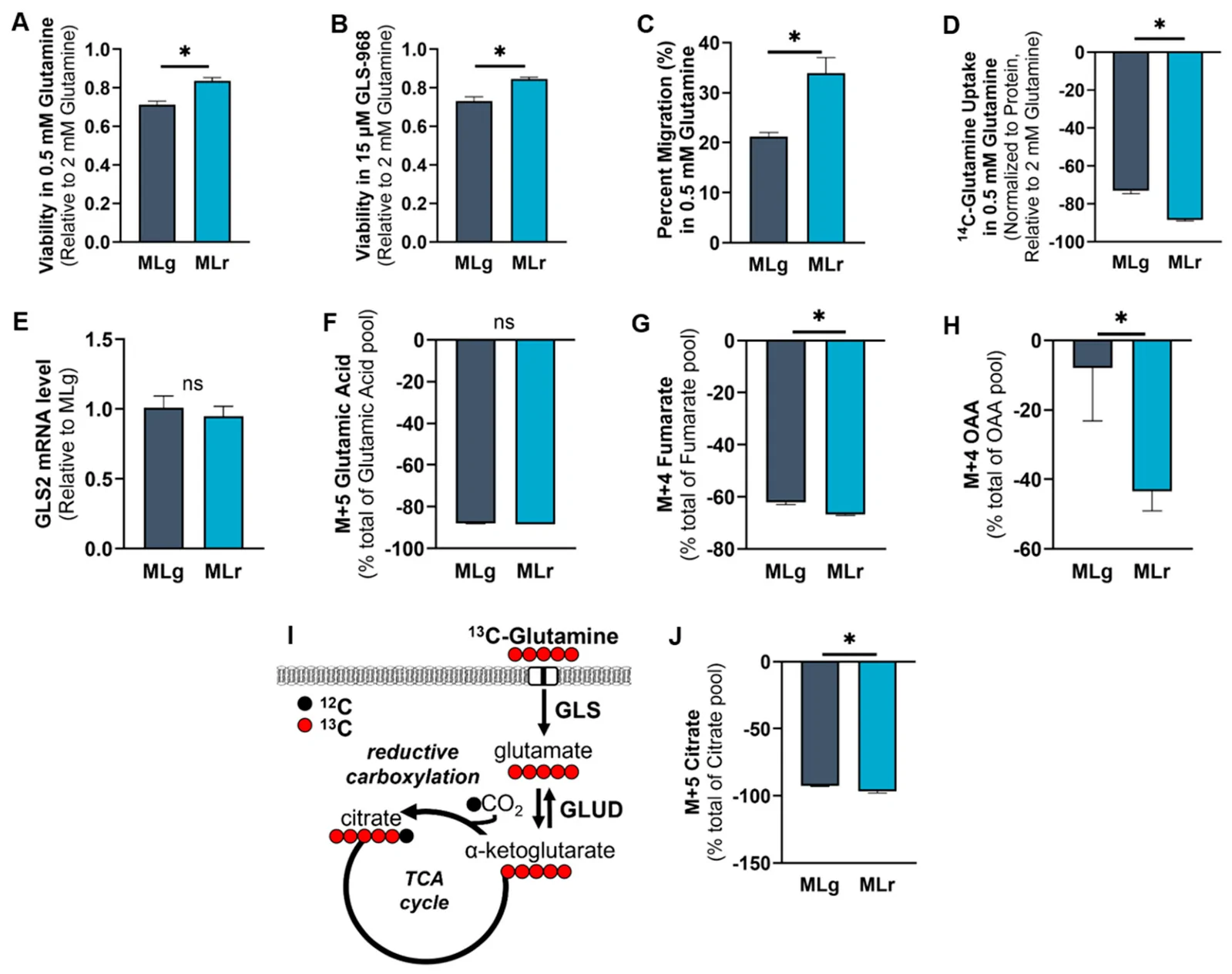
Adaptability to lower glutamine concentrations. (**A**) Viability of the cell lines after treatment with lower glutamine (0.5 mM) or (**B**) 15 μM GLS-968, glutaminase inhibitor, relative to vehicle (DMSO) for 48 h. (**C**) Percent migration (relative to total cells) in cells treated with 0.5 mM glutamine for 17 h. (**D**) ^14^C-glutamine uptake under low glutamine, normalized to protein. (**E**) mRNA abundance of glutaminase 2 (GLS2) after treatment of 0.5 mM glutamine for 48 h, normalized to 18S and relative to metM-Wnt^Lung^. ^13^C_5_-glutamine incorporation into (**F**) M+5-labeled glutamic acid, (**G**) M+4-labeled fumarate and (**H**) M+4-labeled oxaloacetate (OAA). (**I**) Schematic diagram of ^13^C_5_-glutamine reductive carboxylation flux into (**J**) M+5-labeled citrate; GLS—glutaminase, GLUD—glutamate dehydrogenase. N = 5 biological replicates for viability assay; N = 4 biological replicates for migration, ^14^C-glutamine uptake, gene expression, and metabolic flux analyses. MLg = metM-Wnt^Lung^; MLr = metM-Wnt^Liver^ Asterisk indicates statistical significance (* *p* < 0.05, Student’s *t*-test); ns—not significant.

We investigated the adaptability of lung- and liver-metastasizing TNBC cells to lower glutamine levels (0.5 mM). Results indicated that metM-Wnt^Liver^ cells were more viable than the metM-Wnt^Lung^ cells both when glutamine levels were 0.5 mM and when glutamine to glutamate conversion is inhibited (GLS-968, 15 μM) (Figure 3A,B, MTT assay). Consistent with these results, trypan blue cell counting showed significantly higher proliferation in metM-Wnt^Liver^ cells (98.0%) than metM-Wnt^Lung^ cells (88.4%) under low glutamine levels. Similarly, metM-Wnt^Liver^ cells’ migration is higher in lower glutamine levels (Figure 3C), suggesting that the metM-Wnt^Liver^ cells are more adaptable than metM-Wnt^Lung^ cells to both low glutamine and when glutamine metabolism is inhibited by GLS-968. Interestingly, ^14^C-glutamine cellular uptake was significantly reduced in both metastatic cell lines under low glutamine as compared to 2 mM glutamine levels, but metM-Wnt^Liver^ cells showed greater reduction in glutamine uptake than metM-Wnt^Lung^ cells (Figure 3D). Moreover, under low glutamine condition, *Gls2* level was similar between the metastatic cell lines (Figure 3E), and intracellular flux of ^13^C_5_-glutamine into the TCA cycle decreased relative to ^13^C_5_-glutamine flux at 2 mM glutamine levels (Figure 3F–J). Despite comparable levels of M+5-labeled glutamic acid (Figure 3F), the initial product of glutamine catabolism between the metastatic cell lines, metM-Wnt^Liver^ cells exhibited significantly lower enrichment of downstream metabolites, M+4-labeled fumarate and M+4-labeled oxaloacetate (Figure 3G,H), as compared to the metM-Wnt^Lung^ cells. In addition, M+5-labeled citrate, indicative of reductive glutamine carboxylation, was significantly lower in the metM-Wnt^Liver^ cells than the metM-Wnt^Lung^ cells (Figure 3I,J). These results suggest that the metM-Wnt^Liver^ cells reduced glutamine flux into the TCA cycle to a greater extent than the metM-Wnt^Lung^ cells under glutamine deprivation.

### 3.5. Liver-Metastasizing TNBC Cells, but Not Lung-Metastasizing TNBC Cells, Utilize Glutamine for Glutathione Synthesis at Lower Glutamine Levels

**Figure 4:**
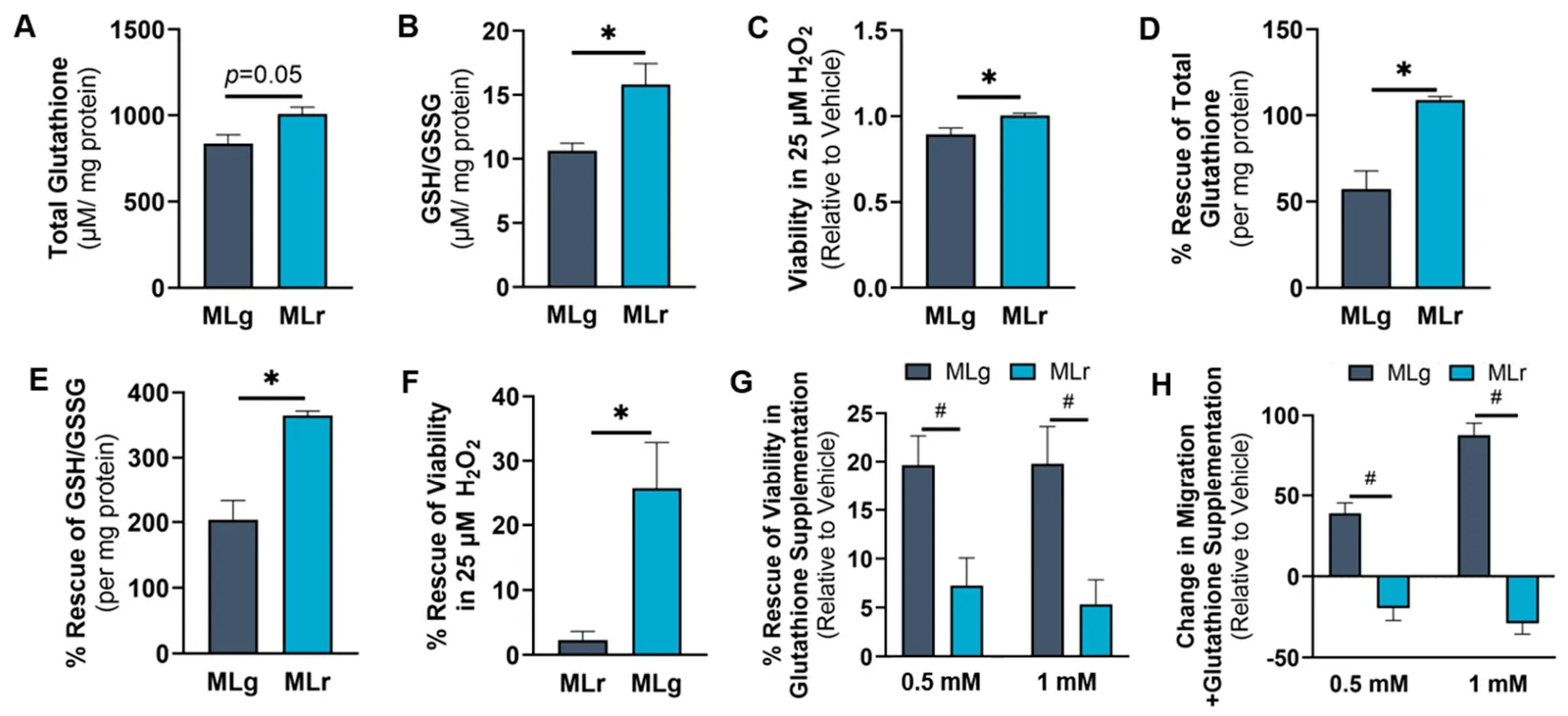
Glutathione levels and oxidative stress protection under lower glutamine concentrations. (**A**) Total glutathione (GSH+GSSG) content and (**B**) ratio of GSH/GSSG under 0.5 mM glutamine for 48 h. (**C**) Viability of the cell lines after co-treatment of 0.5 mM glutamine with 25 μM H_2_O_2_ for 24 h. (**D**) Percent rescue of the total glutathione content and (**E**) ratio of GSH/GSSG after re-supplementation of 2 mM glutamine for 48 h. (**F**) Percent rescue of viability in 25 μM H_2_O_2_ for 24 h after re-supplementation of 2 mM glutamine for 48 h. (**G**) Percent rescue of viability after glutathione supplementation (0.5 mM or 1 mM) in 0.5 mM glutamine for 48 h; no interaction effects. (**H**) Change in migration after glutathione supplementation (0.5 mM or 1 mM) in 0.5 mM glutamine for 17 h; interaction effects: *p* = 0.0014. N = 4 biological replicates for glutathione and migration assays; N = 5 biological replicates for viability assay. MLg = metM-Wnt^Lung^; MLr = metM-Wnt^Liver^. Asterisk indicates statistical significance (* *p* < 0.05, Student’s *t*-test); pound sign indicates statistical significance (# *p* < 0.05, Bonferroni’s multiple comparisons test).

To investigate the reduction in glutamine flux into the TCA cycle in the metM-Wnt^Liver^ cells, we hypothesized that, under glutamine deprivation, metM-Wnt^Liver^ cells may utilize glutamate for glutathione synthesis, thereby limiting the glutamine-derived carbon flux into the TCA cycle. Results showed that the metM-Wnt^Liver^ cells had higher total glutathione and GSH/GSSG, indicative of higher oxidative stress protection capability (Figure 4A,B). Consistent with these, metM-Wnt^Liver^ cells had higher viability both under low glutamine levels (Figure 3A) and with oxidative stress (H_2_O_2_, Figure 4C). Similarly, supplementation of 2 mM glutamine significantly rescued the total glutathione and GSH/GSSG ratio of the metM-Wnt^Liver^ cells, relative to the metM-Wnt^Lung^ cells, as well as viability upon H_2_O_2_ treatment (Figure 4D–F). Interestingly, there was significantly lower rescue in metM-Wnt^Liver^ cells and higher rescue in metM-Wnt^Lung^ cells with supplementation of glutathione (0.5 and 1 mM) (Figure 4G; no interaction effects). Furthermore, significant interaction effects were observed on cell migration upon glutathione supplementation (Figure 4H), indicating that the cell migration of the metastatic cell lines responds differently to glutathione supplementation under lower glutamine levels. Specifically, glutathione treatment (0.5 and 1 mM) increased migration and resulted in significantly greater rescue in metM-Wnt^Lung^ cells compared with metM-Wnt^Liver^ cells (Figure 4H). Whether glutathione synthesis is functionally required for adaptation under glutamine deprivation remains to be fully determined; however, our results suggest that enhanced glutathione synthesis is one plausible adaptive mechanism utilized by metM-Wnt^Liver^ cells when glutamine is limiting, supported by glutathione abundance, redox ratio, and rescue data, which are associated with enhanced viability, migration, and oxidative stress protection in metM-Wnt^Liver^ cells in low glutamine conditions.

### 3.6. Liver-Metastasizing TNBC Cells, but Not Lung-Metastasizing TNBC Cells, Utilize Glucose to Support Viability at Lower Glutamine Levels

**Figure 5:**
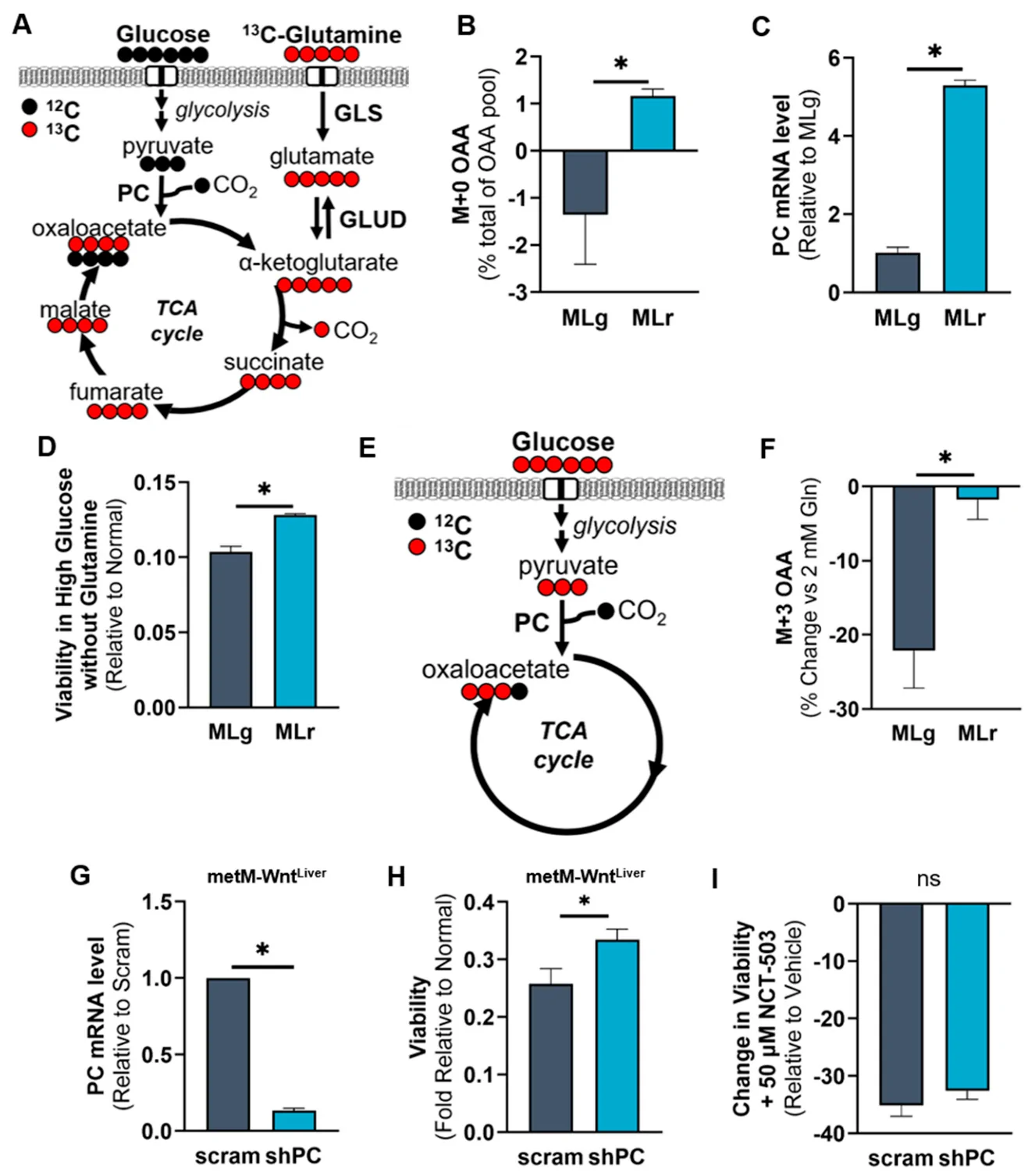
Glucose metabolism in low glutamine concentrations. (**A**) Schematic diagram of glucose flux into (**B**) M+0 unlabeled oxaloacetate (OAA); PC—pyruvate carboxylase, GLS—glutaminase, GLUD—glutamate dehydrogenase. mRNA abundance of (**C**) pyruvate carboxylase (PC) after treatment of 0.5 mM glutamine for 48 h, normalized to 18S and relative to metM-Wnt^Lung^. (**D**) Viability of the cell lines after treatment with 25 mM glucose without glutamine for 48 h. (**E**) Schematic diagram of ^13^C_6_-glucose incorporation into (**F**) M+3-labeled oxaloacetate (OAA) under low glutamine levels. (**G**) mRNA abundance of PC following constitutive PC silencing in metM-Wnt^Liver^ cells. (**H**) Viability of scram and shPC metM-Wnt^Liver^ cells under low glutamine for 48 h. (**I**) Change in viability of scram and shPC metM-Wnt^Liver^ cells after treatment with phosphoglycerate dehydrogenase (PHGDH) inhibitor, NCT-503 (50 μM), under low glutamine for 48 h. N = 4 biological replicates for metabolic flux, and gene expression analyses; N = 5 biological replicates for viability assay. MLg = metM-Wnt^Lung^; MLr = metM-Wnt^Liver^. Asterisk indicates statistical significance (* *p* < 0.05, Student’s *t*-test); ns—not significant.

We further observed that the pool of unlabeled (M+0) oxaloacetate, indicative of pyruvate carboxylase (PC) activity, during ^13^C_5_-glutamine tracing was significantly higher in the metM-Wnt^Liver^ cells compared to the metM-Wnt^Lung^ cells (Figure 5A,B), suggesting a possible compensatory role of glucose metabolism through PC as an adaptive mechanism to further overcome glutamine deprivation in the metM-Wnt^Liver^ cells. Consistent with this, the mRNA level of *Pc* was higher under low glutamine (Figure 5C), and viability is higher upon exposure to excessive glucose (25 mM) without glutamine in the metM-Wnt^Liver^ cells (Figure 5D). Further, metM-Wnt^Liver^ cells had higher maintenance of glucose incorporation into M+3-labeled oxaloacetate under low glutamine levels (Figure 5E,F), suggesting the role of PC in the glutamine adaptability of metM-Wnt^Liver^ cells. However, constitutive silencing of PC in the metM-Wnt^Liver^ cells (shPC, Figure 5G) improved the viability under low glutamine levels (Figure 5H). We hypothesized that the silencing of PC results in the diversion of glucose carbons to alternative pathways upstream of pyruvate to support the adaptability of the metM-Wnt^Liver^ cells. Inhibition of phosphoglycerate dehydrogenase (PHGDH; a key enzyme for de novo serine synthesis from glucose-derived 3-phosphoglycerate) with NCT-503 (50 μM) abrogated the improved viability of the shPC metM-Wnt^Liver^ cells (Figure 5I). These results suggest that the metM-Wnt^Liver^ cells maintain PC activity and utilize glucose, which is associated with enhanced oxidative stress protection, viability and migration under glutamine deprivation, and we further propose that metM-Wnt^Liver^ cells can plausibly divert glucose into de novo serine synthesis under glutamine deprivation when PC activity is impaired.

## 4. Discussion

In this study, we investigated glutamine metabolism and adaptive responses in TNBC cells with distinct metastatic preferences for the lungs (metM-Wnt^Lung^) versus liver (metM-Wnt^Liver^). Our results demonstrate that metM-Wnt^Liver^ cells exhibit greater metabolic adaptability to varying glutamine conditions, maintaining higher viability and migration than metM-Wnt^Lung^ cells. Furthermore, we identified key mechanisms associated with this adaptability under both higher and lower glutamine levels. While glutamine metabolism has been widely studied in cancer, our work provides new mechanistic insights into how these TNBC cells with preferential metastasis to lungs or liver differentially metabolize and adapt to varying glutamine levels.

Previously, triple-negative 4T1-derived lung-homing breast cancer cells grown in 4 mM glutamine displayed higher oxidative glutamine metabolism than the liver-homing counterpart [26]. Similarly, we previously reported that the metM-Wnt^Lung^ cells have higher glutamine flux under both 2 mM and 4 mM glutamine levels, but were unable to adapt to high glutamine levels due to ammonia toxicity as compared to their nonmetastatic counterpart [27]. In the current study, we report that the metM-Wnt^Lung^ cells have higher glutamine flux under 2 mM glutamine levels as compared to the metM-Wnt^Liver^ cells. Our results suggest that upregulated glutamine flux of metM-Wnt^Lung^ cells increase the vulnerability of the cells to excessive metabolic waste products from glutamine catabolism, including ammonia [28]. In contrast, the metM-Wnt^Liver^ cells upregulated enzymes involved in ammonia utilization, thus plausibly providing protection from increased glutamine metabolism. It has been demonstrated that ammonia is reassimilated into central metabolism to maximize nitrogen utilization through the generation of amino acids and stimulation of cell growth and proliferation in MCF7 and T47D breast cancer cells [28]. In the current study, the upregulation of enzymes such as *Asns* and *Cad* suggests that the assimilation of ammonia into different intermediary metabolites may connect with a wide variety of distinct biological processes such as amino acid synthesis [28]. Also, the previous literature suggests that breast cancer cells that metastasize to the liver can acquire the specific metabolic ability of the liver, including ammonia metabolism [29]. Thus, it is possible that the upregulation of ammonia metabolism may be a critical determinant of successful metastasis to the liver, and the absence of this metabolic capability in the metM-Wnt^Lung^ cells results in their inability to successfully metastasize to the liver metabolic niche.

Intriguingly, inhibiting glutamine pathways does not uniformly reduce viability, even in glutamine-dependent TNBC cells [18]. For instance, inhibiting GLS activity with CB-839 in several TNBC cell lines demonstrated that CB-839-resistant cells were less glutaminolytic, and instead increased mitochondrial fatty acids’ oxidation compared to CB-839-sensitive cells [19]. Clinical trials on CB-839 has been in phase I-II clinical trials for cancer therapy, including in TNBC [30]. However, resistance to CB-839 arises because inhibition of glutamine catabolism can be bypassed when cells are supplied with exogenous TCA intermediates such as α-KG or oxaloacetate. Under these conditions, increased glucose contribution to the TCA cycle compensates for the loss of glutamine-derived carbon, resulting in overall TCA metabolite levels remaining largely unchanged [30]. Additionally, glutamine is considered to be the rate-limiting factor in glutathione synthesis, and its depletion has been associated with decreased intracellular glutathione levels [31]. Our findings suggest that metM-Wnt^Liver^ cells exhibit a distinct adaptive response by utilizing glutamine-derived glutamate for glutathione synthesis. Additionally, replenishment of oxaloacetate through PC provides an alternative anaplerotic pathway when glutamine is scarce [32]. Consistent with this, our study shows that the more glutaminolytic metM-Wnt^Lung^ cells were more sensitive to glutamine deprivation, whereas metM-Wnt^Liver^ cells maintained higher cell viability under both glutamine deprivation and GLS inhibition. This was accompanied by the maintenance of high PC, supporting the concept that glucose metabolism via PC contributes to the metabolic flexibility of metM-Wnt^Liver^ cells under glutamine-limited conditions. In addition to supporting anaplerotic reactions, glucose can serve as a precursor for de novo serine synthesis. The one-carbon units from serine, a non-essential amino acid and a third key nutrient in cancer metabolism after glucose and glutamine, support purine and thymidine synthesis, methylation reactions, and NADPH production for cellular antioxidant defense [33]. The observed utilization of de novo serine synthesis with shPC metM-Wnt^Liver^ cells is potentially caused by pyruvate accumulation resulting in the accumulation of glycolytic intermediates such as 2-phosphoglycerate, which is a positive allosteric regulator of PHGDH [34]. It has been previously shown that deprivation of glutamine-activated de novo serine biosynthesis pathway and glutathione synthesis from serine is critical for cell survival, proliferation and apoptosis control through redox balance [35]. Consistent with this, our work suggests glutamine use for glutathione synthesis and a plausible compensatory role of glucose, PC, and serine synthesis in supporting adaptations for cell viability and oxidative stress protection in metM-Wnt^Liver^ cells under low glutamine levels.

## 5. Conclusions

Altogether, we report that metM-Wnt^Liver^ cells sustain viability and migration during periods of metabolic perturbation, such as (i) upregulating enzymes that utilize excess ammonia under high glutamine conditions; and (ii) suggesting glutamine use for glutathione synthesis and supporting glucose-derived anaplerosis via PC, which are associated with enhanced cell viability and oxidative stress protection under low glutamine conditions. Conversely, the metM-Wnt^Lung^ cells appear more dependent on extracellular glutamine, making them more vulnerable to glutamine deprivation and less capable of adapting to glutamine excess. Our study highlights the metabolic heterogeneity of these metastatic TNBC cells and underscores the potential role of glutamine metabolism in site-specific adaptations. Further investigation is warranted to determine whether these metabolic phenotypes are generalizable across other breast cancer subtypes with site-tropic tendencies toward the lung or liver, and whether these observed phenotypes are determinants of adaptation. Nonetheless, defining these differential metabolic dependencies provides an important clinical foundation for developing site-tailored metabolic interventions. Therapeutic strategies that simultaneously restrict glucose and glutamine metabolism may be necessary to disrupt the adaptive capacity of TNBC cells and limit metastatic progression specifically to the lung and liver.

## Figures and Tables

**Table 1:** Primer sequences.

GENE (Mouse)	SEQUENCE
18S	Forward: 5′-ATC CCT GAG AAG TTC CAG CA-3′
	Reverse: 5′-CCT CTT GGT GAG GTC GAT GT-3′
Glutaminase 2 (*Gls2*)	Forward: 5′-CAG AGG GAC AGG AGC GTA TC-3′ Reverse: 5′-TTC TTT CGG AAT GCC TGA GTC-3′
Glutamate dehydrogenase (*Glud*)	Forward: 5′-CCC AAC TTC TTC AAG ATG GTGG-3′ Reverse: 5′-AGA GGC TCA ACA CAT GGT TGC-3′
Asparagine synthetase (*Asns*)	Forward: 5′-CACAAGGCGCTACAGCAAC-3′ Reverse: 5′-CCAGCATACAGATGGTTTTCTCG-3′
Carbamoyl-phosphate synthetase 2 (*Cad*)	Forward: 5′-GGGGAAGTGGTGTTTCAGACC-3′ Reverse: 5′-CGTAGTTGCCGATGAGAGGAT-3′
Pyruvate carboxylase (*Pc*)	Forward: 5′-TCA CCA GTG ACT CTG TCA AAC-3′ Reverse: 3′-GAC CAG GTC CAC ATC TGT AAT-3′

## Data Availability

The original contributions presented in this study are included in the article. Further inquiries can be directed to the corresponding author.

## Abbreviations

TNBC	Triple-negative breast cancer
TCA	Tricarboxylic acid
GSH	Reduced glutathione
GSSG	Oxidized glutathione
PC	Pyruvate carboxylase
PHGDH	Phosphoglycerate dehydrogenase
GLS	Glutaminase
DMSO	Dimethyl sulfoxide
DMEM	Dulbecco’s Modified Eagle Medium
FBS	Fetal bovine serum
PBS	Phosphate-buffered saline
GLUD	Glutamate dehydrogenase
ASNS	Asparagine synthetase
CAD	Carbamoyl-phosphate synthetase 2
